# Prevalence and genetic evolution of porcine reproductive and respiratory syndrome virus in commercial fattening pig farms in China

**DOI:** 10.1186/s40813-024-00356-y

**Published:** 2024-01-22

**Authors:** Chao Li, Jing Zhao, Wansheng Li, Hu Xu, Bangjun Gong, Qi Sun, Zhenyang Guo, Jinhao Li, Lirun Xiang, Yan-dong Tang, Chaoliang Leng, Qian Wang, Jinmei Peng, Guohui Zhou, Huairan Liu, Tongqing An, Xuehui Cai, Zhi-Jun Tian, Hongliang Zhang

**Affiliations:** 1 State Key Laboratory for Animal Disease Control and Prevention, Harbin Veterinary Research Institute, Chinese Academy of Agricultural Sciences, CAAS, No. 678 Haping Road, Xiangfang District, Harbin, 150001 China; 2 Henan Key Laboratory of Insect Biology in Funiu Mountain, Henan Provincial Engineering Laboratory of Insects Bioreactor, China-UK-NYNU-RRes Joint Laboratory of Insect Biology, Nanyang Normal University, Nanyang, 473061 China

## Abstract

**Background:**

To investigate the prevalence and evolution of Porcine Reproductive and Respiratory Syndrome Virus (PRRSV) at commercial fattening pig farms, a total of 1397 clinical samples were collected from a single fattening cycle at seven pig farms in five provinces of China from 2020 to 2021.

**Results:**

The RT‒PCR results revealed that PRRSV was present on all seven farms, and the percentage of PRRSV-positive individuals was 17.54–53.33%. A total of 344 partial NSP2 gene sequences and 334 complete ORF5 gene sequences were obtained from the positive samples. The statistical results showed that PRRSV-2 was present on all seven commercial fattening farms, and PRRSV-1 was present on only one commercial fattening farm. A total of six PRRSV-2 subtypes were detected, and five of the seven farms had two or more PRRSV-2 subtypes. L1.8 (L1C) PRRSV was the dominant epidemic strain on five of the seven pig farms. Sequence analysis of L1.8 (L1C) PRRSV from different commercial fattening pig farms revealed that its consistency across farms varied substantially. The amino acid alignment results demonstrated that there were 131 aa discontinuous deletions in NSP2 between different L1.8 (L1C) PRRSV strains and that the GP5 mutation in L1.8 (L1C) PRRSV was mainly concentrated in the peptide signal region and T-cell epitopes. Selection pressure analysis of GP5 revealed that the use of the PRRSV MLV vaccine had no significant episodic diversifying effect on L1.8 (L1C) PRRSV.

**Conclusion:**

PRRSV infection is common at commercial fattening pig farms in China, and the percentage of positive individuals is high. There are multiple PRRSV subtypes of infection at commercial fattening pig farms in China. L1.8 (L1C) is the main circulating PRRSV strain on commercial fattening pig farms. L1.8 (L1C) PRRSV detected at different commercial fattening pig farms exhibited substantial differences in consistency but similar molecular characteristics. The pressure on the GP5 of L1.8 (L1C) PRRSV may not be directly related to the use of the vaccines.

**Supplementary Information:**

The online version contains supplementary material available at 10.1186/s40813-024-00356-y.

## Background

Porcine reproductive and respiratory syndrome (PRRS) is caused by porcine reproductive and respiratory syndrome virus (PRRSV), a single-stranded RNA enveloped virus of the genus *Betaarterivirus*, family *Arteriviridae*, and order *Nidovirales* [1]. Clinically, the virus causes reproductive disorders in sows and severe respiratory diseases in piglets [2]. PRRSV can be divided into two species, *Betaarterivirus suid 1* (PRRSV-1) and *Betaarterivirus suid 2* (PRRSV-2), which share 60% nucleotide (nt) identity at the whole-genome level [1]. PRRSV encodes at least 10 open reading frames. The NSP2 protein is frequently used in molecular epidemiological investigations due to its characteristic amino acid deletion and insertion [3–5], and as ORF5 exhibits high genetic diversity, it is widely used for phylogenetic analysis [3, 6].

According to phylogenetic tree analysis, PRRSV-1 is classified into subtype 1 (global), subtype 1 (Russia), subtypes 2 and 3, and PRRSV-2 is classified into lineages 1–11 [6–10]. Since the PRRSV strain CH-1a (L8.7/L8E, CH-1a-like PRRSV) was reported in China in 1996, sublineage 5.1/5A (L5.1, ATCC-VR2332-like PRRSV); sublineage 3.5 (L3.5, QYYZ-like PRRSV); sublineage 8.7/8E (L8.7/L8E, HP-PRRSV-like PRRSV), which has a deletion of 1+29 amino acids in the NSP2 gene of PRRSV compared to CH-1a-like PRRSV; sublineage 1.8/1C (L1.8/L1C, NADC30-like PRRSV); and sublineage 1.5/1A (L1.5/L1A NADC34-like PRRSV) in PRRSV-2 have appeared successively in China [11, 12]. Although PRRSV-2 is predominant in China, PRRSV-1 isolates (subtype 1, global) have also existed in China for more than twenty years [13]. Long-term epidemiological surveillance has shown that PRRSV epidemic strains alternate [14]. With the development of the breeding industry in China, an increasing number of commercial fattening pig farms have gradually been established. However, the prevalence of PRRSV at commercial fattening pig farms in China is less well described, and the effects of that prevalence on the evolution of PRRSV at commercial fattening pig farms in China have not been fully elucidated. In this study, PRRSV monitoring was carried out on seven commercial fattening pig farms in China for a single fattening cycle (approximately 150 days) to clarify the prevalence of PRRSV on different farms and the molecular characteristics of the main strains.

## Materials and methods

### Farm information

The geographical location and breeding scale of the seven commercial fattening pig farms are presented in Table 1. Each farm was well located and had professional veterinary practitioners and professional management staff. All the fattening pig farms adopted an all-in, all-out fully enclosed management model; personnel and materials entering and leaving the area were disinfected.

**Table 1:** Pig farms information

Name	Farming scale	Vaccination	Monitoring time	Death rate (%)	Sample size	Number of positive samples	Positive rate (%)	Subtypes
Heilongjiang A farm	1000	No vaccination	2020	1.60	180	38	36.58	L1.8(L1C) PRRSV
Heilongjiang B farm	3400	CH-1R MLV^a^	2020	12.09	283	82	29.33	L1.8(L1C) PRRSV, L1.5(L1A) PRRSV, L8.7(L8E, HP-PRRSV-like PRRSV), L8.7(L8E, CH-1a-like PRRSV) and L3.5(QYYZ-like PRRSV)
Heilongjiang C farm	4000	HuN4-F112 MLV^b^	2020–2021	5.55	239	107	44.77	L1.8(L1C) PRRSV and L8.7(L8E, HP-PRRSV-like PRRSV)
Henan farm	500	No vaccination	2020	4.00	132	38	28.79	PRRSV-1, L8.7(L8E, HP-PRRSV-like PRRSV), L1.8(L1 C) PRRSV and L5.1(L5A, ATCC-VR2332-like PRRSV)
Xinjiang farm	1400	No vaccination	2020	16.86	236	91	38.56	L1.8(L1C) PRRSV and L8.7(L8E, HP-PRRSV-like PRRSV)
Hebei farm	2000	No vaccination	2020–2021	24.50	270	144	53.33	L1.8(L1C) PRRSV and L1.5(L1A) PRRSV
Hubei farm	2000	TJM MLV^c^	2020–2021	2.49	57	10	17.54	L8.7(L8E, HP-PRRSV-like PRRSV)

^a^Each about 60-day-old piglet were vaccinated intramuscularly 1 dose (2 ml) with CH-1R MLV

^b^Each about 60-day-old piglet were vaccinated intramuscularly 1 dose (2 ml) with HuN4-F112 MLV

^c^Each about 15-day-old piglet were vaccinated intramuscularly 0.5 dose (1 ml) with TJM MLV

### Sample collection and genome sequencing

Serum, lung and submaxillary lymph node samples were collected proportionately by veterinary professionals at each farm, and laboratory tests were performed every 15 days during the study period. Tissue sample processing and sequence primer synthesis were performed according to previous methods [14–16]. The positive samples were sequenced using the Sanger method. Tissue samples were homogenized in Dulbecco’s modified Eagle’s medium (Gibco) using a TissueLyser II (Qiagen) for RNA extraction. The samples were then stored at − 80 °C until analysis. After reverse transcription, three pairs of primers designed by our laboratory for PRRSV NSP2, ORF5, and ORF7 were used for detection. Positive samples were amplified using TaKaRa LA Taq^®^ to obtain target bands. After fragment recovery using a commercial kit, the PMD-18T vector was ligated, followed by transformation and identification of the bacterial suspension. Once the identification was confirmed, three bacterial liquid samples were taken and sent to a company for sequencing. The three obtained sequences were assembled into the final sequence using SeqMan software.

### Phylogenetic and genomic analysis

Multiple sequence alignments were performed using MAFFT version 7 in BioAider V1.527 [17, 18] with default parameters and manually adjusted in MEGA6. Deduced amino acid sequences were aligned with ClustalW sequences with Lasergene software. Phylogenetic trees were constructed in MEGA 6.0 by the neighbour‒joining method with a bootstrap value of 1000 replicates and with the Kimura two-parameter substitution model. All the Chinese L1.8 (L1C) PRRSV reference sequences were obtained from the NCBI library and were available as of December 31, 2022. FigTree and EvolView were used to visualize the phylogenetic tree.

### Recombination analysis

The preliminary identification of possible recombination events in ORF5 was performed using RDP4 [19].

### Selective pressure analysis

Methods focused on selection in a short period or at individual sites have been very successful when applied to real data analysis [20]. An analysis of the selection pressure acting on the branch of L1.8 (L1C) PRRSV was conducted using the Datamonkey webserver (http://www.datamonkey.org/) with adaptive branch-site random effects likelihood (aBSREL), which permits selective pressure on sequences, quantified by the ω ratio, to vary among both codon sites and individual branches in the phylogeny [21–23]. If a branch has an inferred ω > 1 and an LRT *p* value < 0.05, the virus is estimated to evolve under episodic positive (diversifying) selection at that branch. Analysis of stress-related GP5 selection sites on different farms was performed using fixed effects likelihood (FEL) models [24]. If a site has an inferred *p* value < 0.05, the virus is estimated to have evolved under episodic positive selection at that site.

### Statistical analysis

Statistical analysis and visualization of the data were performed using GraphPad Prism 8.0 (San Diego, CA, USA).

## Results

### PRRSV positive rate at commercial fattening pig farms

**Fig. 1:**
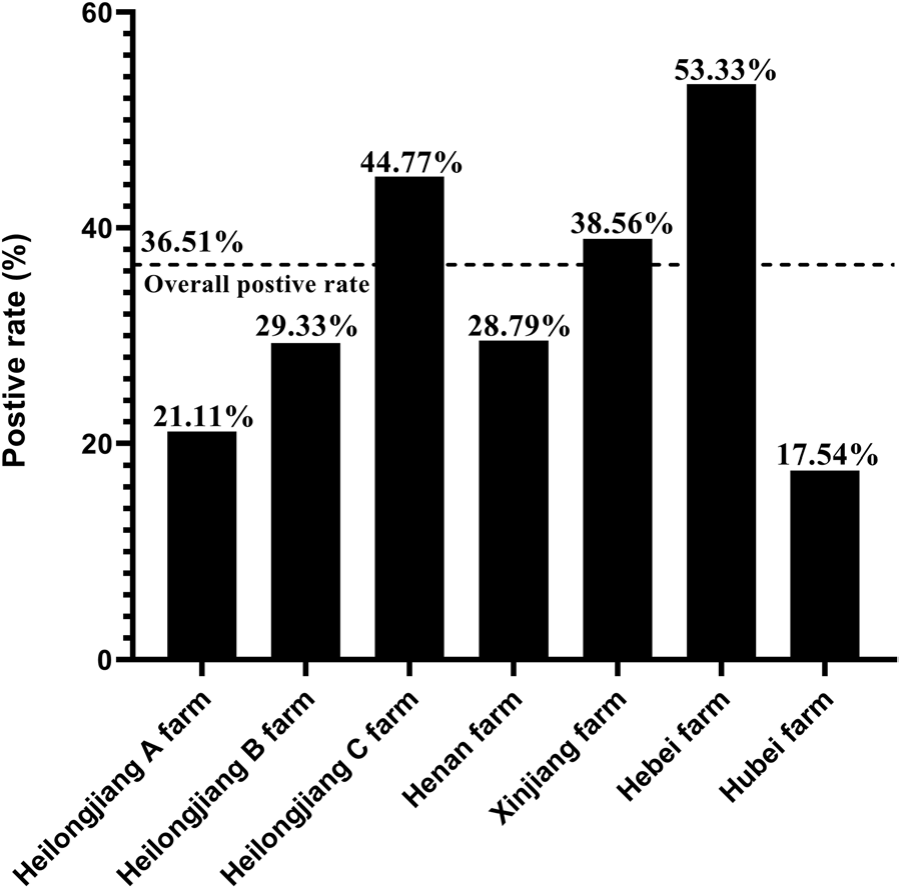
Positive rate of PRRSV in commercial pig fattening farms. The dotted line represents the overall PRRSV-positive rate for the seven commercial farms

To investigate the prevalence of PRRSV at commercial fattening pig farms, seven farms of different sizes in five provinces were randomly selected for PRRSV monitoring during a fattening cycle (Table 1). PRRSV was detected on all seven commercial fattening farms (Heilongjiang A farm, Heilongjiang B farm, Heilongjiang C farm, Xinjiang farm, Hebei farm, Henan farm and Hubei farm), and a total of 510 positive samples were detected (Additional file 1: Table S1), for an overall positive rate of 36.51% (Fig. 1). The percentages of PRRSV antigen-positive individuals at the individual commercial fattening pig farms were 17.54% (Hubei farm), 21.11% (Heilongjiang A farm), 28.79% (Henan farm), 29.33% (Heilongjiang B farm), 38.56% (Xinjiang farm), 44.77% (Heilongjiang C farm) and 53.33% (Hebei farm) (Fig. 1); moreover, the percentage of PRRSV-positive individuals at the 3 commercial fattening farms, namely, the Heilongjiang C Farm, Xinjiang Farm and Hebei Farm, was greater than the overall percentage of PRRSV-positive individuals (36.51%). These results indicate that PRRSV is widely distributed on commercial fattening pig farms and has a high antigen-positive rate in China.

### Statistics of PRRSV subtypes at commercial fattening pig farms

The PRRSV antigen-positive samples were sequenced, and a total of 344 partial NSP2 sequences, 334 complete ORF5 sequences and 63 ORF7 sequences were obtained (Additional file 1: Table S1). Phylogenetic analysis and aa alignment revealed that 497 samples (97.26%) were PRRSV-2 and 14 samples (2.74%) were PRRSV-1; these samples all belonged to the BJEU06-1-like group of subtype 1 (global) (Additional file 2: Fig. S1–S3). There were six PRRSV-2 subtypes on the farms: L1.5 (L1A) PRRSV, L1.8 (L1C) PRRSV, L3.5 PRRSV, L5.1 (L5A) PRRSV, L8.7 (L8E, CH-1a-like PRRSV) and L8.7 (L8E, HP-PRRSV-like PRRSV). The results showed that PRRSV subtypes are complex in commercial fattening pigs in China.

**Fig. 2:**
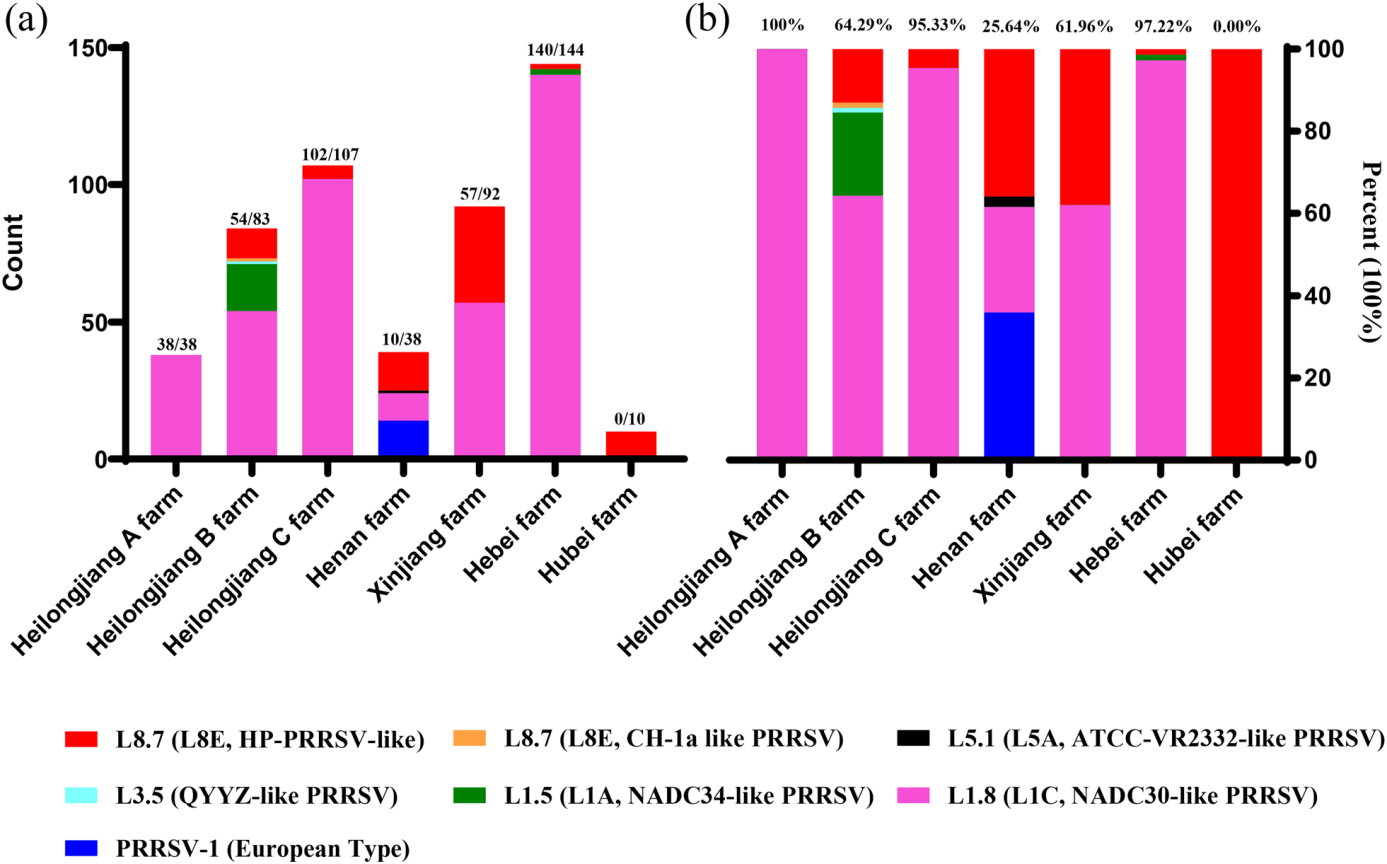
Statistical analysis of PRRSV subtypes in seven commercial fattening pig farms. **a** The number of subtypes and the number of samples in each subtype. **b** Proportion of PRRSV subtypes. Subtypes are represented by different colours. The positive samples and percentage of L1.8 (L1C) PRRSV is shown at the top of the bar chart

To explore the number of different PRRSV subtypes detected at the individual farms, statistical analyses were performed on the data from the seven commercial fattening farms (Fig. 2). Both the Heilongjiang A farm (L1.8/L1C PRRSV) and the Hubei farm [L8.7 (L8E, HP-PRRSV-like PRRSV)] had only one subtype strain. There were five PRRSV subtypes on the Heilongjiang B farm, L1.8 (L1C) PRRSV, L1.5 (L1A) PRRSV, L8.7 (L8E, HP-PRRSV-like PRRSV), L8.7 (L8E, CH-1a-like PRRSV) and L3.5 PRRSV, and four PRRSV subtypes on the Henan farms, subtype 1 (global) of PRRSV-1, L8.7 (L8E, HP-PRRSV-like PRRSV), L1.8 (L1C) PRRSV and L5.1 (L5A) PRRSV, which we previously reported [15, 16]. Two PRRSV subtypes, L1.8 (L1C) PRRSV and L8.7 (L8E, HP-PRRSV-like PRRSV), were found on the Heilongjiang C farm. There were two subtypes, L1.8 (L1C) PRRSV and L8.7 (L8E, HP-PRRSV-like PRRSV), at the Xinjiang farm. The L1.8 (L1C) PRRSV, L1.5 (L1A) PRRSV and L8.7 (L8E, HP-PRRSV-like PRRSV) PRRSV subtypes were found on the Hebei farm. The results showed that only two commercial fattening pig farms were infected with a single subtype of PRRSV and that the other five farms were infected with multiple PRRSV subtypes, which indicates that multiple types of PRRSV coexist at most individual commercial fattening pig farms.

### Main circulating strain at commercial fattening pig farms

The data for the PRRSV subtypes detected at the seven commercial fattening pig farms revealed that 401 samples were L1.8 (L1C) PRRSV (80.85%), 77 samples were L8.7 (L8E, HP-PRRSV-like PRRSV, 15.40%), 19 samples were L1.5 (L1A) PRRSV (3.72%), 1 sample was L3.5 PRRSV (0.20%), 1 sample was L5.1 (L5A) PRRSV (0.20%), and 1 sample was L8.7 (L8E, CH-1a-like PRRSV, 0.20%). Moreover, the L1.8 (L1C) PRRSV and L8.7 (L8E, HP-PRRSV-like PRRSV) strains were detected in large numbers and existed on six of the pig farms, while L1.5 (L1A) PRRSV, L3.5 PRRSV, L5.1 (L5A) PRRSV and L8.7 (L8E, CH-1a-like PRRSV) were detected on only one or two of the pig farms. To determine the main viral subtypes that are prevalent at commercial fattening pig farms, statistical analyses were performed on the data from the seven farms (Fig. 2). The results showed that L1.8 (L1C) PRRSV accounted for the highest percentage of PRRSV-positive individuals at five of the commercial pig farms: the Heilongjiang A farm, 100% (38/38); the Heilongjiang B farm, 64.29% (54/83); the Heilongjiang C farm, 95.32% (102/107); the Xinjiang farm, 61.96% (57/92); and the Hebei farm, 97.22% (140/144). Overall, L1.8 (L1C) was the predominant PRRSV strain among the Chinese commercial fattening pig farms from 2020 to 2021.

### NSP2 deletion characteristics of L1.8 (L1C) PRRSV at different commercial fattening farms

**Fig. 3:**
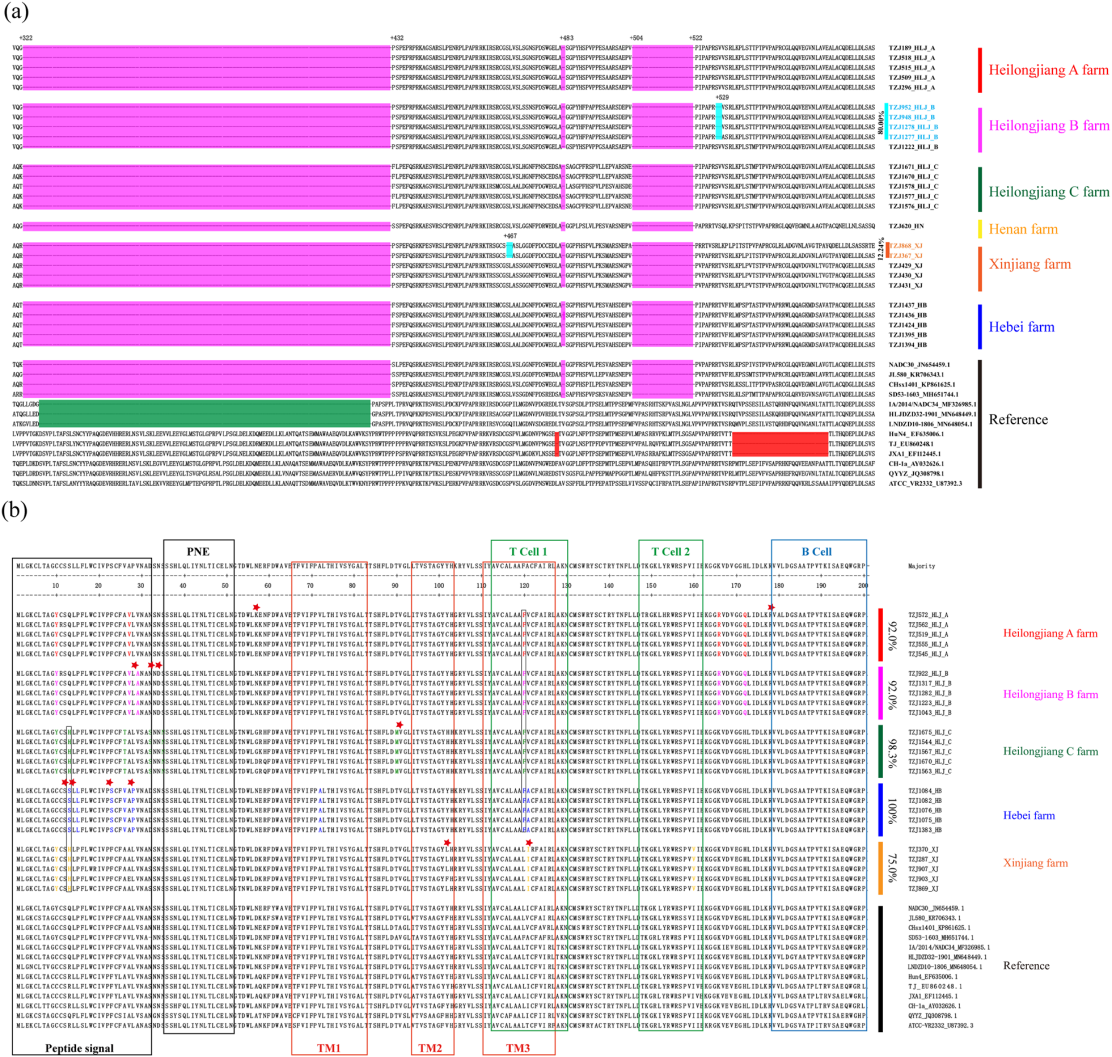
Molecular characteristics of NSP2 and GP5 of L1.8(L1C) PRRSV. **a** NSP2 deletion characteristics of L1.8(L1C) PRRSV on pig farms. The positions marked in the figure represent positions of the NSP2 amino acid sequence and refer to the position of ATCC VR2332. Purple indicates the NADC30-like PRRSV 131 aa characteristic discontinuous deletion. Additional aa deletions are indicated in sky blue, and their proportions are indicated on the right side of the sequence. **b** Alignment of full-length GP5 aa sequences of part-positive L1.8(L1C) PRRSV strains. Upper grey rectangles show antigenic regions (PNE—principal neutralizing epitope), and lower grey rectangles show biologically significant regions (Signal sequence; TM—transmembrane region). The rightmost digit of the aa sequence is the proportion of GP5 sequences with L1.8(L1C) molecular characteristics identical on a single farm. The aa mutation of L1.8(L1C) on a given pig farm are coloured differently than the reference strain. Positive selection sites are marked with red fivepointed stars

On the farms where L1.8 (L1C) PRRSV was detected (the Heilongjiang A farm, Heilongjiang B farm, Heilongjiang C farm, Hebei farm, Henan farm and Xinjiang farm), all the NSP2 of L1.8 (L1C) PRRSV had 111 + 1 + 19 aa deletions, which indicates that 111 + 1 + 19 remains a molecular signature unique to L1.8 (L1C) PRRSV (Fig. 3a). Moreover, in contrast to what was shown for NSP2 of ATCC-VR2332, we identified two novel distinct NSP2 deletion patterns, with two additional consecutive aa deletions in 467 and 468 of L1.8 (L1C) PRRSV at the Heilongjiang B farm and two additional consecutive aa deletions in 529 and 530 of L1.8 (L1C) PRRSV at the Xinjiang farm (Fig. 3a). Additionally, L1.8 (L1C) PRRSV, which has two new characteristics, accounted for 80% (44/55) and 12.24% (6/49) of the L1.8 (L1C) PRRSV strains in the Heilongjiang B farm and the Xinjiang farm, respectively (Fig. 3a).

### ORF5 phylogenetic tree analysis of L1.8 (L1C) PRRSV at different commercial fattening farms

**Fig. 4:**
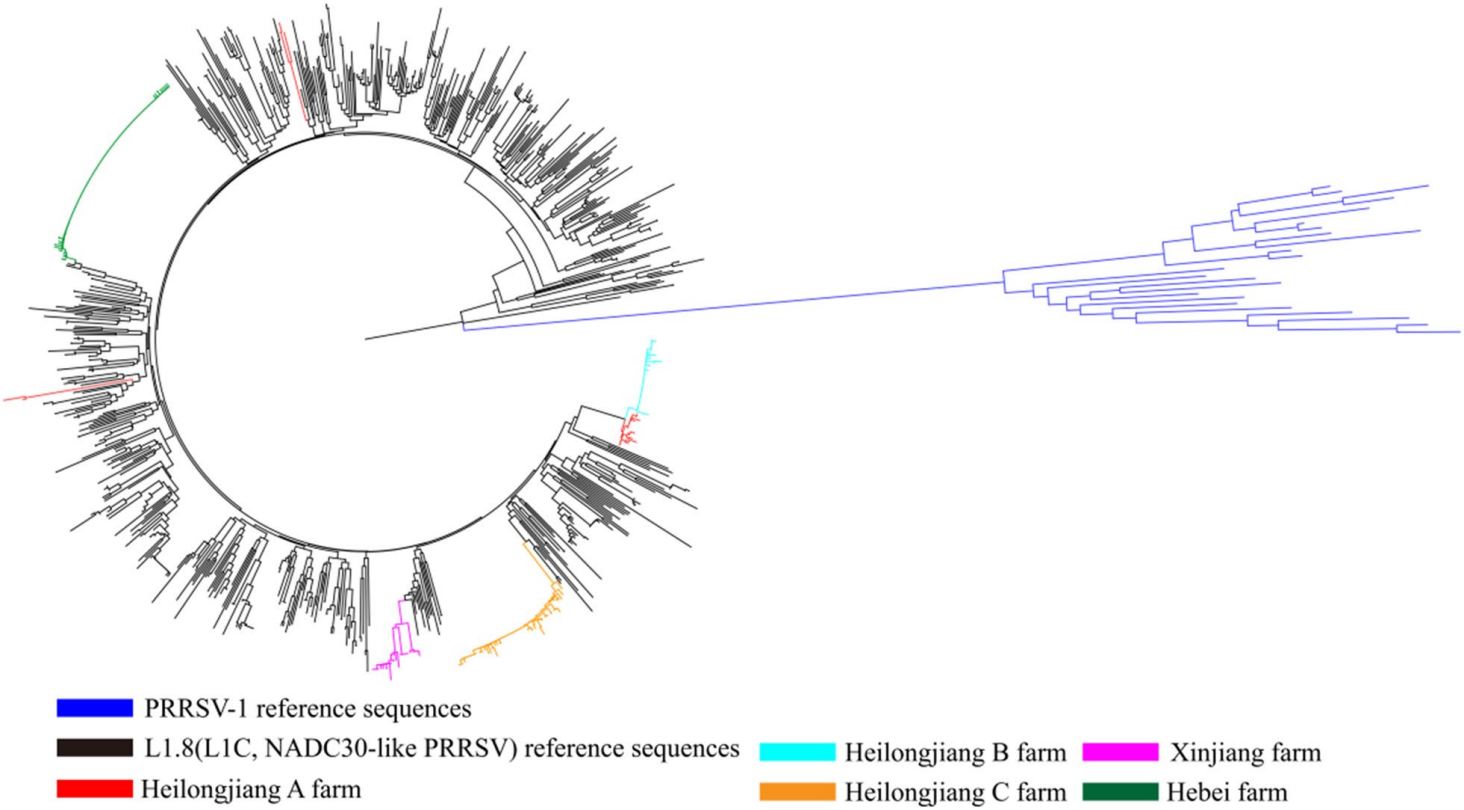
Phylogenetic analysis based on the ORF5 gene of L1.8(L1C) PRRSV. Neighbour-joining trees based on ORF5 and bootstrap values at the nodes are based on 1000 replicates. L1.8(L1C) PRRSVS from different commercial fattening pig farms are represented in different colours

To study the origin and evolution of L1.8 (L1C) PRRSV on pig farms, sequencing data for all L1.8 (L1C) PRRSV strains from China were downloaded from the NCBI library (up to December 31, 2022) and used as the background (reference sequence) to construct phylogenetic trees for analysis. ORF5 phylogenetic tree analysis revealed that L1.8 (L1C) PRRSV in a single commercial fattening pig farm (Heilongjiang B farm, Heilongjiang C farm, Hebei farm and Xinjiang farm) was mainly concentrated in the same branch (Fig. 4). Moreover, we also detected the presence of different branches of L1.8 (L1C) PRRSV on the Heilongjiang A farm (Fig. 4). The L1.8 (L1C) PRRSV from the Heilongjiang A farm was divided into three different branches with large genetic distances between the branches (Fig. 4).

To determine whether the presence of the detected branch was caused by recombination, we performed recombination analysis on L1.8 (L1C) PRRSV sequences from each commercial fattening pig farm via RDP4. The results showed no significant recombination signals.

### Consistency analysis of NSP2 and ORF5 against L1.8 (L1C) PRRSV at different commercial fattening farms

The nt consistency of L1.8 (L1C) PRRSV NSP2 at different individual pig farms was analysed, and the results showed that the Heilongjiang C farm (97.9–100%) and the Hebei farm (97.3–100%) had greater nt consistency than did the other three farms (Heilongjiang A farm: 81.3–99.8%; Heilongjiang B farm: 74.8–100%; Xinjiang farm: 78.5–100%) (Table 2). Based on the consistency analysis of ORF5, the Heilongjiang B farm (98.3–100%), Heilongjiang C farm (98.0–100%) and Hebei farm (98.8–99.9%) had greater nt and aa consistency than did the Xinjiang farm (90.3–100%) and Heilongjiang A farm (87.1–100%) (Table 2).

**Table 2:** Nucleotide identity analysis for NSP2 and ORF5 of L1.8(L1C) PRRSV and L8.7 (L8E, HP-PRRSV-like PRRSV)

Names of pig farms	NSP2/ORF5 of L1.8(L1C) PRRSV (%) and NSP2/ORF5 of L8.7(L8E, HP-PRRSV-like PRRSV) (%)						
	Heilongjiang A farm	Heilongjiang B farm	Heilongjiang C farm	Xinjiang farm	Hebei farm	Henan farm	Hubei farm
Heilongjiang A farm	(81.3–99.8/87.1–100) and (–/–)	(66.4–98.9/88.5–98.00) and (–/–)	(62.6–84.9/87.6–91.5) and (–/–)	(65.6–85.2/86.4–91.7) and (–/–)	(73.6–84.8/88.1–90.8) and (–/–)	(–/–) and (–/–)	(–/–) and (–/–)
Heilongjiang B farm	(–/–) and (–/–)	(74.8–100/98.3–100)and (88.4–100/98.3–100)	(60.7–84.7/89.0–91.7) and (-/98.2–99.8)	(63.9–84.5/89.3–91.3) and (-/96.7–99.3)	(64.1–84.8/88.7–90.7) and (none/98.3–99.8)	(–/–) and (89.3–99.4/98.3–99.8)	(–/–) and (82.6–97.0/98.3–99.5)
Heilongjiang C farm	(–/–) and (–/–)	(–/–) and (–/–)	(97.9–100/98.0–100) and (-/98.5–99.7)	(63.2–84.1/88.8–91.5) and (none/96.9–99.2)	(63.9–85.2/87.8–89.8) and (none/98.7–99.7)	(–/–) and (none/99.3–99.8)	(–/–) and (none/98.7–99.7)
Xinjiang farm	(–/–) and (–/–)	(–/–) and (–/–)	(–/–) and (–/–)	(78.5–100/90.3–100) and (none/95.5–100)	(65.3–88.3/88.3–90.0) and (none/96.4–99.2)	(–/–) and (none/96.9–99.7)	(–/–) and (none/97.0–99.2)
Hebei farm	(–/–) and (–/–)	(–/–) and (–/–)	(–/–) and (–/–)	(–/–) and (–/–)	(97.3–100/98.8–99.9) and (none/98.2)	(–/–) and (none/98.0–99.8)	(–/–) and (none/98.3–99.0)
Henan farm	(–/–) and (–/–)	(–/–) and (–/–)	(–/–) and (–/–)	(–/–) and (–/–)	(–/–) and (–/–)	(–/–) and (100/98.7–100)	(–/–) and (none/98.0–99.8)
Hubei farm	(–/–) and (–/–)	(–/–) and (–/–)	(–/–) and (–/–)	(–/–) and (–/–)	(–/–) and (–/–)	(–/–) and (–/–)	(–/–) and (93.3–100/99.2–100)

According to the nucleotide consistency of ORF5 and NSP2, the nucleotide consistency within pig farms was greater than that among pig farms for both L1.8 (L1C) PRRSV and L8.7 (L8E, HP-PRRSV-like PRRSV) (Table 2). The nucleotide consistency of the NSP2 and ORF5 sequences of L1.8 (L1C) PRRSV was much lower than that of L8.7 (L8E, HP-PRRSV-like PRRSV) among pig farms, which indicates that the genetic evolution of L1.8 (L1C) PRRSV among fattening pig farms in China has been more complex. Notably, the nucleotide concordance of L8.7(L8E) PRRSV was high both within a single farm and among different farms; however, L1.8 (L1C) PRRSV showed lower nucleotide concordance within some pig farms as well as among different pig farms (Table 2).

### Molecular characteristics of L1.8 (L1C) PRRSV between vaccinated farms and unvaccinated farms

To investigate the genetic diversity of L1.8 (L1C) PRRSV between vaccinated farms and unvaccinated farms (Additional file 3: Table S2), multiple sequence alignment was conducted via the ClustalW method using Lasergene of the DNASTAR package. ORF5 sequence alignments showed no deletions or insertions, but substitutions were frequently observed regardless of whether the farm used the vaccine (Fig. 3b). Compared with the positions at which the ORF5 aa was replaced in previously reported strains (NADC30: JN654459.1, JL580: KR706343.1 and CHsx1401: KP861625.1), the L1.8 (L1C) PRRSV strains from both vaccinated and unvaccinated farms shared the same molecular characteristics. L1.8 (L1C) PRRSV variations from the pig farms mentioned above were mainly concentrated in peptide signals, and an analysis of GP5 protein epitopes revealed that the variation in the GP5 protein was mainly concentrated in T-cell epitopes (Fig. 3b).

To investigate whether there was a selective pressure for L1.8 (L1C) PRRSV GP5 in the abovementioned commercial fattening pig farms caused by the use of the PRRSV commercial vaccine (Additional file 3: Table S2), this study performed a selection stress test on all L1.8 (L1C) PRRSV branches from farms with and without commercial vaccines. Selection pressure analysis of the phylogeny suggested that GP5 evolved mostly under near-neutrality conditions, regardless of vaccine use (Table 3). After confirming that the use of vaccines had no obvious selective pressure on the GP5 strain of the L1.8 (L1C) PRRSV clade, we further explored whether the aa of the GP5 strain of L1.8 (L1C) PRRSV faced additional selective pressure on farms using commercial vaccines. Three and one positive selection sites were identified on the Heilongjiang B farm and Heilongjiang C farm, respectively, where commercial vaccine was used, and 2, 2, 4, and 0 sites were identified as positive selection sites on the Heilongjiang A farm, Xinjiang farm, Hebei farm, and Henan farm, respectively, where no vaccine was used (Table 3, Additional file 2: Fig. S4). It is not surprising that no positive selection sites were found at Henan farm because only one GP5 sequence was detected at Henan farm, preventing further analysis of selection pressure. In addition, the results showed that the above selective pressure sites were evenly distributed within the GP5 protein and had no obvious direct relationship with the use of commercial vaccines. Thus, we found that the use of the PRRSV-MLV vaccine had no additional influence on the GP5 concentration among the circulating PRRSV strains on five farms.

**Table 3:** Selective pressure analysis of GP5 of L1.8(L1C) PRRSV

Vaccination	Name	Selective pressure analysis				
		aBSREL		FEL		
		ω	*p* value	Sites of positive selection	alpha = beta	*p* value
Vaccined	Heilongjiang B farm	ω1 = 0.224(100%)	1	29	0.892	0.0135
				33	0.749	0.004
				34	1.013	0.002
	Heilongjiang C farm	ω1 = 0.0(100%)	1	91	2.927	0.005
		ω1 = 0.431(100%)	1			
No vaccined	Heilongjiang A farm	ω1 = 0.0716 (95%)	0.0002	57	3.334	0.0578
		ω2 = 36.6 (4.9%)				
		ω1 = 0.283 (100%)	1	178	1.193	0.076
		ω1 = 0.308 (100%)	1			
	Xinjiang farm	ω1 = 0.113 (100%)	1	102	1.98	0.069
				120	1.764	0.0043
	Hebei farm	ω1 = 0.000112 (91%)	0.149	13	1.633	0.0018
		ω2 = 3.68 (9.0%)				
				15	0.607	0.0583
				23	0.873	0.0593
				28	0.76	0.0625
	Henan farm	ω1 = 1.00 (100%)	0	\	\	\

aBSREL, (ω: non-synonymous to synonymous substitutions ratio or dN/dS, class Site classification at (0 > 1 and LRT *p* value < 0.05; FEL, alpha = beta: the rate estimate under the neutral model, *p* value: asymptotic *p* value for evidence of selection, i.e. beta &neq; alpha, class Site classification at *p *<  = 0.1

## Discussion

### Highly positive rates of PRRSV at commercial fattening pig farms in China

With the development of industrialization and global trade, the impact of PRRSV on the pig industry across the world is deepening [25]. Commercial fattening pig farms of different sizes in different provinces were randomly selected in this study to determine the prevalence of PRRSV at commercial fattening pig farms in China. In this study, we collected 1397 samples from seven different commercial fattening farms across five provinces in China from 2020 to 2021. In previous reports [26–28], sampling was usually conducted only at a certain point in time. To more accurately determine the prevalence of PRRSV at individual commercial fattening farms, we monitored the prevalence of PRRSV on seven farms for a period of one fattening cycle (approximately 150 days). PRRSV was detected during this period, and the percentage of PRRSV-positive pigs at the pig farms ranged from 17.54 to 53.33%. Although this study effectively monitored PRRSV in seven commercial fattening pig farms in different provinces, it may not accurately reflect the prevalence of PRRSV in a single province due to the limited number of commercial fattening pig farms in different provinces.

### Multiple subtypes of PRRSV coexpress on commercial fattening pig farms in China

Long-term monitoring of PRRSV will be helpful for more comprehensively elucidating the infection situation at commercial fattening pig farms, providing insights of great significance for the prevention and control of PRRSV. The coexistence of multiple PRRSV subtypes has always been a problem faced by the global pig industry [29–31]. Previous studies have demonstrated that PRRSV-2 has long been considered the predominant species in China, cocirculating with PRRSV-1 [11]. Six PRRSV-2 subtypes, including L8.7 (L8E, CH-1a-like PRRSV), L5.1 (L5A), L3.5, L8.7 (L8E, HP-PRRSV-like PRRSV), L1.8 (L1C) and L1.5 (L1A), have been successively reported in China since 1996 [3, 11]. Our laboratory previously conducted PRRSV surveillance on a pig farm for up to 4 years, and the results showed the presence of the above multiple PRRSV-2 subtype strains at Chinese pig farms [14]. In this study, for the first time, the PRRSV subtypes at seven commercial fattening pig farms were reported. Multiple PRRSV subtypes were found on most of these Chinese commercial fattening pig farms. Coinfection with multiple PRRSV subtypes is highly dangerous because PRRSV fragments can be exchanged between subtypes to form strains with different virulence [32, 33]. However, the relationship between PRRSV recombination and virulence changes still requires additional conclusive evidence.

### L1.8 (L1C) PRRSV is the predominant strain found on commercial fattening pig farms in China

NADC30, first isolated in 2008 in the United States, has the “111 + 1 + 19” deletion characteristic of NSP2 [7, 34, 35]. Subsequently, an increasing number of L1.8 (L1C) PRRSV strains emerged in the U.S. and became the dominant epidemic strain in 2010 [9, 10]. The first L1.8 (L1C) PRRSV was detected in China in 2012 [36, 37], and it became the dominant strain in 2016 [38, 39]. Studies have shown that the incidence of emerging lineages of PRRSV peaks within approximately 4.5 years, and the prevalence of dominant strains changes approximately every three years [10]. L1.5 (L1A) PRRSV became the dominant strain in the United States in 2016 [10]. It is not clear whether the main circulating strains of PRRSV have changed at commercial fattening farms in China. Most of the PRRSV-2 strains obtained in this study were L1.8 (L1C) PRRSV (78.47%, 401/510), which had the widest range (6/7, 85.7%) and accounted for the largest positive proportion on five of the six pig farms, indicating that L1.8 (L1C) PRRSV remains the predominant circulating strain at commercial fattening pig farms.

### L1.8 (L1C) PRRSV showed substantial differences in consistency at different commercial fattening pig farms

On the basis of the nt consistency analysis (of NSP2 and ORF5) results for the Heilongjiang C and Hebei farms, the L1.8 (L1C) PRRSV at these two farms appears to have evolved from a single-strain infection. Despite the different patterns of NSP2 deletion in L1.8 (L1C) PRRSV, there was high consistency in ORF5 in L1.8 (L1C) PRRSV on the Heilongjiang B farm; therefore, we believe that the L1.8 (L1C) PRRSV at this farm also evolved from a single strain and that its NSP2 gene was partially absent during the process. One surprise was the low consistency of L1.8 (L1C) PRRSV at the Xinjiang farm (90.3–100%), with L1.8 (L1C) PRRSV occurring on the same phylogenetic tree branch, indicating that this difference may be from the rapid evolution of a single strain over a short period. L1.8 (L1C) PRRSV had low nt consistency within the Heilongjiang A farm (NSP2: 81.3–99.8%, ORF5: 87.1–100%), and phylogenetic tree analysis revealed multiple branches, indicating that the low consistency of L1.8 (L1C) PRRSV was due to the presence of multiple L1.8 (L1C) PRRSV strains. Diversity analysis of L1.8 (L1C) PRRSV strains on farms showed that the degree of differentiation of single strains over a short period (a fattening cycle of approximately 150 days) was still low, but the consistency of L1.8 (L1C) PRRSV among pig farms was very low. In previous long-term surveillance of pig farms, a single L1.8 (L1C) PRRSV strain rapidly evolved into a single branch within two years, and the nt identity within the single branch rapidly decreased [14]. Therefore, the greater consistency of L1.8 (L1C) PRRSV at a single pig farm may be due to the short duration of the fattening period. Based on the above results, the low consistency of L1.8 (L1C) PRRSV among the different fattening pig farms suggested that L1.8 (L1C) PRRSV has been strongly mutated in China and has spread in different regions. The consistency of L1.8 (L1C) PRRSV among pig farms varies significantly, which poses new challenges for current commercial vaccines.

### Similar molecular characteristics among different commercial fattening pig farms

The PRRSV monitoring results from the seven pig farms in five different provinces made it feasible to conduct a multidimensional analysis of L1.8 (L1C) PRRSV. The deletion pattern of PRRSV NSP2 is one of the main pieces of evidence in the epidemiological investigation of PRRSV. The deletion pattern of 131 aa in NSP2 was specific to the L1.8 (L1C) PRRSV strain, but we also found different additional deletions in NSP2 from L1.8 (L1C) PRRSV at different pig farms. However, the effect of this new NSP2 deletion on PRRSV has not been determined. ORF5 is an ideal candidate for phylogenetic tree construction, not only because it exhibits marked genetic variation within its relatively short length [6] but also because it encodes the major envelope glycoprotein GP5, which has many important functional areas that were explored in detail in previous studies [40–42]. The peptide signal region is critical for PRRSV infection [40, 43], and we found many aa mutations in this region of L1.8 (L1C) PRRSV. The positive selection site in GP5 can be driven by the host immune response [43].

### Role of commercialized vaccines in the prevention and control of PRRSV

When PRRSV was monitored on commercial fattening pig farms using commercial vaccines (Hubei farm, Heilongjiang B farm and Heilongjiang C farm), we found 1, 5 and 10 strains of the same subtype as the vaccine strains on the three farms, respectively Additional file 3: Table S2, Additional file 2: Fig. S5–S6). The TJM-MLV vaccine used on the Hubei farm has an extra 120 aa deletion in NSP2 [44], but this feature was not found in the NSP2 sequences detected on this farm (Additional file 2: Fig. S6). The vaccines used on the other two farms did not have typical characteristic markers, so it is unclear whether those strains were vaccine-related strains. The efficacy of vaccines in clinical use is difficult to quantify because it involves many factors, such as differences in the virulence of circulating strains, immunization procedures, and farm management [33, 36, 45]. The death rates of the three commercial vaccinated farms were 12.09% (Heilongjiang B farm, Classical-PRRSV MLV), 5.55% (Heilongjiang C farm, HP-PRRSV-MLV) and 2.49% (Hubei farm, HP-PRRSV-MLV) (Additional file 3: Table S2), which showed that HP-PRRSV-MLV may provide protection against PRRSV. The positive selection site in GP5 can be driven by the host immune response (Music and Gagnon, 2010). In PRRSV-2, positive selection pressure acts on amino acid sites potentially associated with immune escape through glycan shielding [46]. Selection pressure analysis indicated that selection for situational diversification along the L1.8 (L1C) PRRSV phylogenetic tree was rare on the farms tested, regardless of vaccine use. This analysis is often used to assess the fitness of viruses in different host species [47]. We subsequently used each pig farm as a subset to analyse the selection pressure of the aa of GP5 on L1.8 (L1C) PRRSV, and we did not find any difference between vaccine-using farms and nonvaccine farms in the aa selection pressure analysis of GP5. We therefore concluded that at these five commercial finishing pig farms, the effect of vaccine used on the diversification of L1.8 (L1C) PRRSV GP5 was not significant. This study has certain limitations because increasing evidence suggests that minor proteins such as GP2, GP3, and GP4 interact with host receptors and contain several neutralizing epitopes [48, 49]. This finding may be related to the absence of a relationship between the vaccines and selection pressure in this study.

Because of inconsistent cross-protection, cocirculation of PRRSV variants may cause clinical losses in production systems, challenge the efficacy of vaccination, and often introduce additional complexity in disease control [45, 50]. Biosecurity is arguably the most compelling strategy for tackling these problems. Elimination through herd closure can also be a long, uncertain, and costly process, with herds often taking approximately 41 weeks after an outbreak is detected to start consistently weaning virus-free piglets [51]. The existing biosafety measures still have a long way to go, so until that happens, the best strategy for reducing domestic losses is the sound use of vaccines. The main circulating strains on pig farms should be evaluated first when selecting vaccines for prevention and control because current commercial vaccines have good protective effects against homologous strains [44, 52]. In China, the outbreak of PRRSV has had a significant impact on the economic performance of pig farms, and most of the losses are caused by a decrease in the number of weaned and fattened piglets and an increase in feed costs [53]. Control of the infection is usually performed by a combination of virus monitoring, biosecurity restrictions, herd management measures and vaccination [54]. Currently, commercial vaccines provide partial protection only against L1.8 (L1C) PRRSV [55–58]; therefore, there is an urgent need to develop a commercial vaccine for this disease.

## Conclusions

In summary, the infection rate of PRRSV at commercial fattening farms from 2020 to 2021 was very high, and various types of PRRSV coexisted at Chinese farms. Importantly, L1.8 (L1C) is currently the most prevalent PRRSV strain on individual commercial fattening farms. Both NSP2 and ORF5 of L1.8 (L1C) PRRSV exhibited great differences in consistency but similar molecular characteristics among different pig farms. The selection pressure for L1.8 (L1C) PRRSV GP5 may not be related to vaccine used. This study fills a gap in the PRRSV epidemiological investigations of commercial fattening pig farms in China and provides important data for PRRSV prevention and control in China.

### Supplementary Information

**Additional file 1: Table S1.** Positive sample detection and sequencing information.

**Additional file 2: Fig. S1.** Phylogenetic tree analysis of PRRSVs based on the NSP2 gene. **Fig. S2. **Phylogenetic tree analysis of PRRSVs based on the ORF5 gene. **Fig. S3.** Phylogenetic tree analysis of PRRSVs based on the ORF7 gene. **Fig. S4.** L1.8(L1C) PRRSV sites selection pressure analysis by FEL. **Fig. S5.** Phylogenetic tree analysis of PRRSVs based on the ORF5 gene from vaccine-related pig farms. **Fig. S6.** NSP2 deletion characteristics of L8.7 PRRSV on Hubei farm.

**Additional file 3: Table S2.** Comparison of nucleotide consistency between vaccine - associated strain and vaccine strain or vaccine mother strain NSP2/ORF5.

## Data Availability

The sequences of this study were deposited in GenBank with the accession numbers OQ986023-OQ986366, OQ920558-OQ920890 and OR062460-OR062521. The sequences will be released to public databases when the data or accession numbers appear in print. The sequence data are supplied in the supplementary files.
