# Supplementary material for: Prevalence and genetic evolution of porcine reproductive and respiratory syndrome virus in commercial fattening pig farms in China
Source: Porcine Health Manag. 2024 Jan 22;10:5. doi: 10.1186/s40813-024-00356-y (PMC10801985; doi:10.1186/s40813-024-00356-y)
Supplement: Supplementary file 2 — Additional file 2: Fig. S1. Phylogenetic tree analysis of PRRSVs based on the NSP2 gene. Fig. S2. Phylogenetic tree analysis of PRRSVs based on the ORF5 gene. Fig. S3. Phylogenetic tree analysis of PRRSVs based on the ORF7 gene. Fig. S4. L1.8(L1C) PRRSV sites selection pressure analysis by FEL. Fig. S5. Phylogenetic tree analysis of PRRSVs based on the ORF5 gene from vaccine-related pig farms. Fig. S6. NSP2 deletion characteristics of L8.7 PRRSV on Hubei farm. [file 40813_2024_356_MOESM2_ESM.pdf]

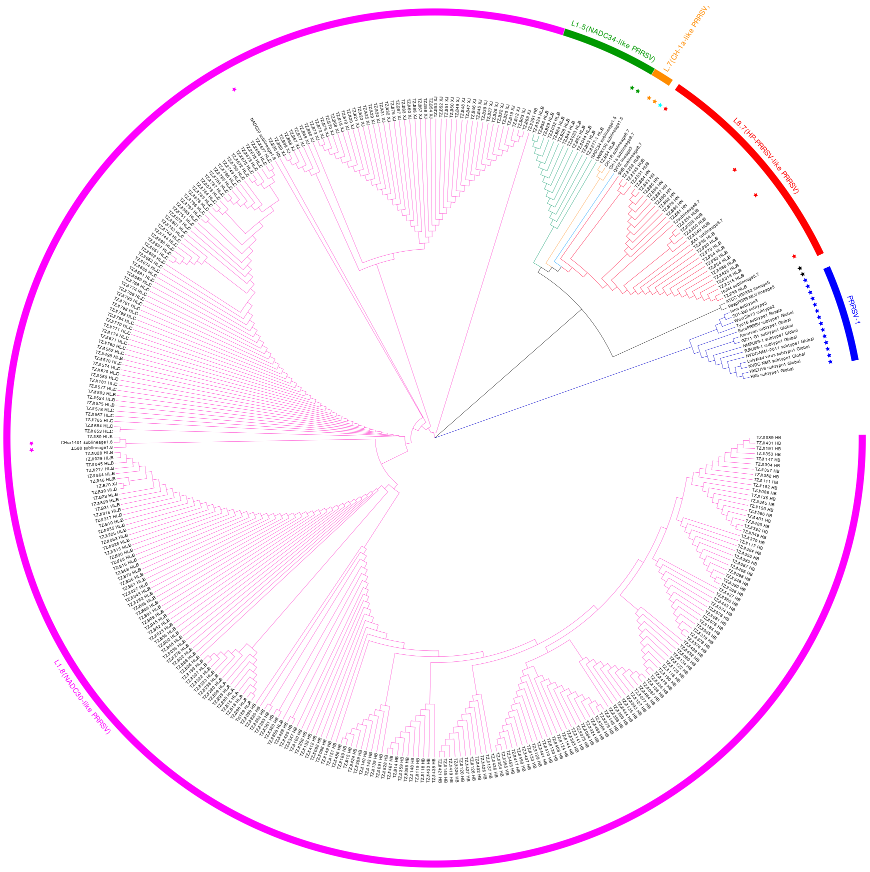

FIG S1. Phylogenetic tree analysis of PRRSVs based on the NSP2 gene. Different subtypes are represented by different colours. Multiple subtype reference strains are labeled with five-pointed stars of different colors.

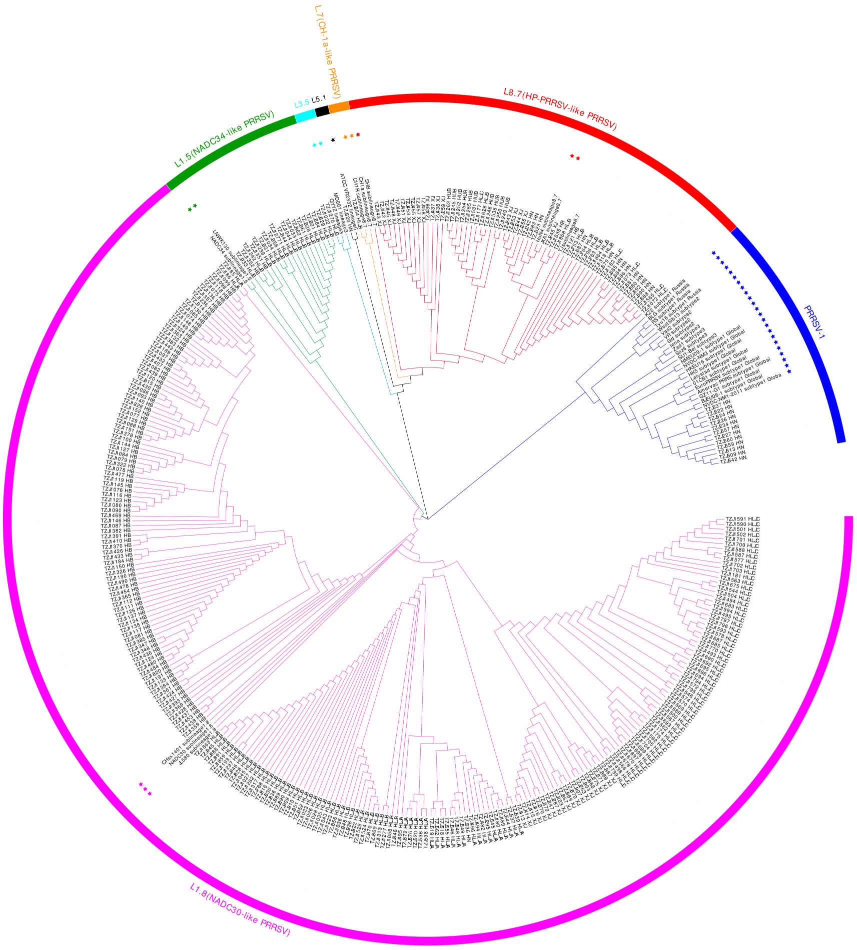

FIG S2. Phylogenetic tree analysis of PRRSVs based on the ORF5 gene. Different subtypes are represented by different colours. Multiple subtype reference strains are labeled with five-pointed stars of different colors

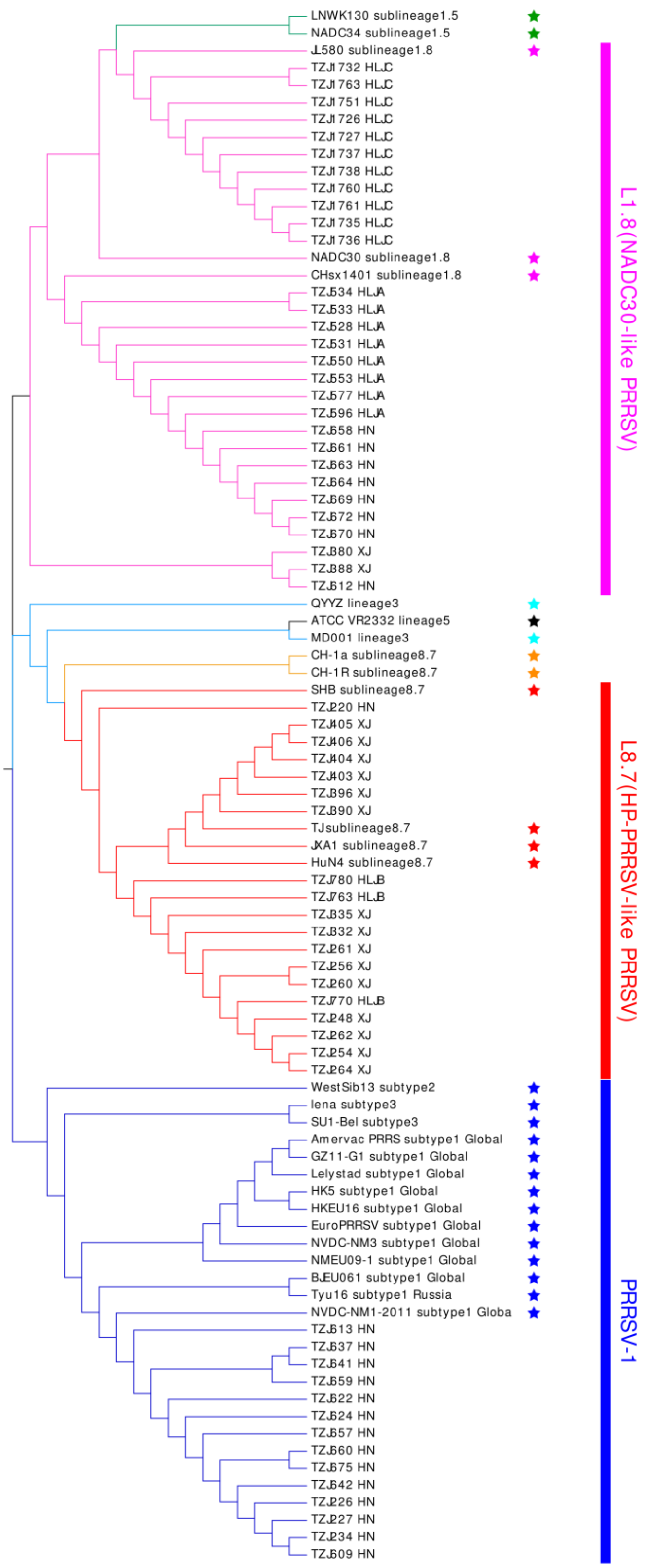

FIG S3. Phylogenetic tree analysis of PRRSVs based on the ORF7 gene. Different subtypes are represented by different colours. Multiple subtype reference strains are labeled with five-pointed stars of different colors

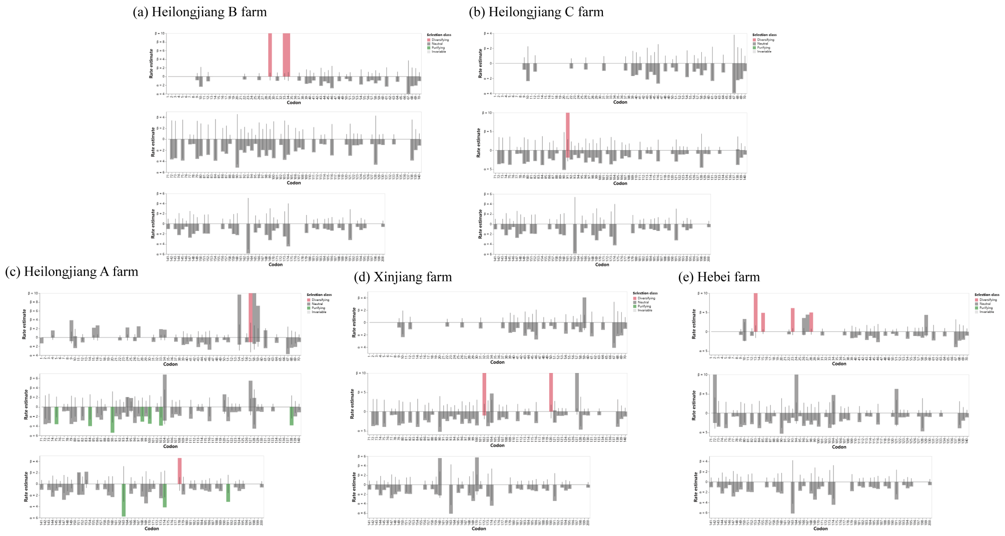

FIG S4. L1.8 PRRSV sites selection pressure analysis by FEL. Maximum likelihood estimates of synonymous (α) and non-synonymous rates (β) at each site shown as bars. The line shows the estimates under the null model (α=β). Estimates above 10 are censored at this value.

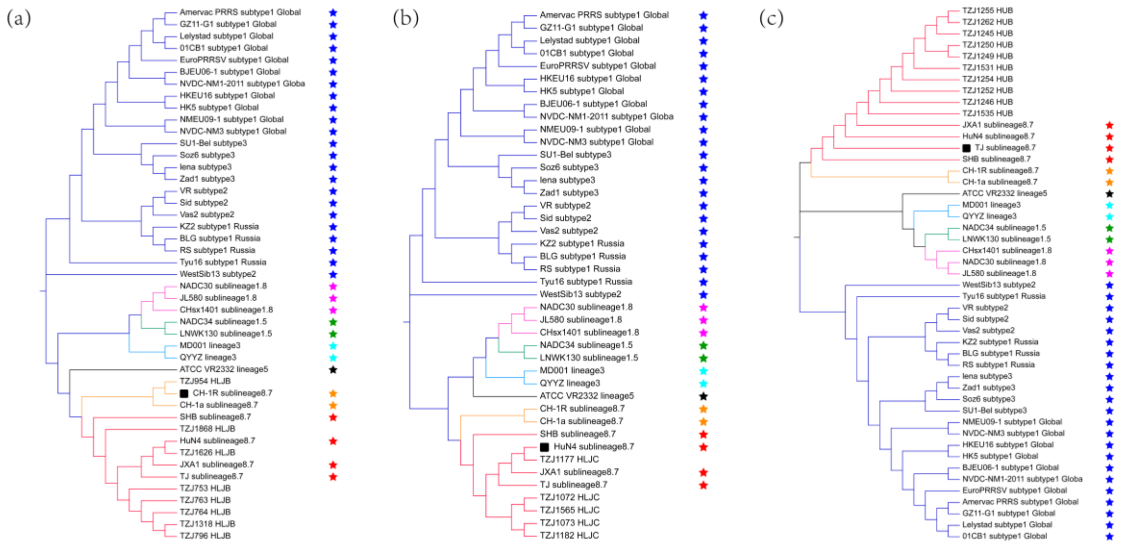

FIG S5. Phylogenetic tree analysis of PRRSVs based on the ORF5 gene from vaccine-related pig farms. Different subtypes are represented by different colours. Multiple subtype reference strains are labeled with five-pointed stars of different colors. Vaccine strains or vaccine parent strains used in pig farms are labeled with black squares. (a) Heilongjiang B farm (b) Heilongjiang C farm (c)Hubei farm

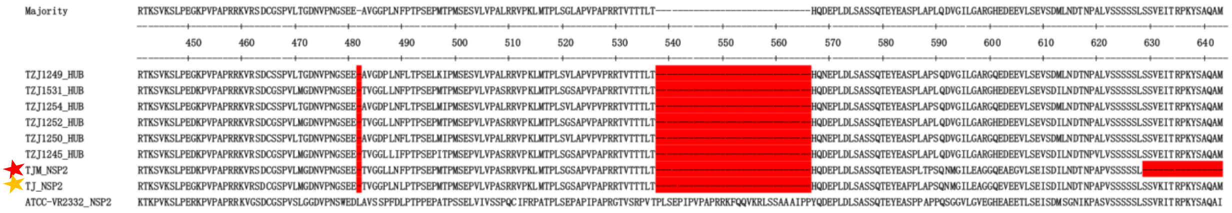

FIG S6. NSP2 deletion characteristics of L8.7 PRRSV on Hubei farm. The vaccine was labeled with a red five-pointed star, and the vaccine parent strain was labeled with a yellow five-pointed star.
