# Supplementary material for: Prevalence and genetic evolution of porcine reproductive and respiratory syndrome virus in commercial fattening pig farms in China
Source: Porcine Health Manag. 2024 Jan 22;10:5. doi: 10.1186/s40813-024-00356-y (PMC10801985; doi:10.1186/s40813-024-00356-y)
Supplement: Supplementary file 3 — Additional file 3: Table S2. Comparison of nucleotide consistency between vaccine - associated strain and vaccine strain or vaccine mother strain NSP2/ORF5. [file 40813_2024_356_MOESM3_ESM.pdf]

TABLE S2. Comparison of nucleotide consistency between vaccine - associated strain and vaccine strain or vaccine mother strain NSP2/ORF5.

Farms	Nucleotide consistency of NSP2/ORF5		
	CH-1R_EU807840.1	HuN4_EF635006.1	TJ_EU860248.1
Heilongjiang B farm	99.00%/98.80%		
Heilongjiang C farm		None/98.7-99.7%	
Hubei farm			93.3-97.3%/99.2-99.5%
